# Adsorption of Uranyl ions on Amine-functionalization of MIL-101(Cr) Nanoparticles by a Facile Coordination-based Post-synthetic strategy and X-ray Absorption Spectroscopy Studies

**DOI:** 10.1038/srep13514

**Published:** 2015-09-10

**Authors:** Jian-Yong Zhang, Na Zhang, Linjuan Zhang, Yongzheng Fang, Wei Deng, Ming Yu, Ziqiang Wang, Lina Li, Xiyan Liu, Jingye Li

**Affiliations:** 1 Shanghai Institute of Technology, Shanghai 200235, P. R. China; 2 CAS Center for Excellence in TMSR Energy System, Shanghai Institute of Applied Physics, Chinese Academy of Sciences, Shanghai 201800, China; 3 Shanghai Synchrotron Radiation Facility, Shanghai Institute of Applied Physics, Chinese Academy of Sciences, Shanghai 201204, China

## Abstract

By a facile coordination-based post-synthetic strategy, the high surface area MIL-101(Cr) nanoparticles was functionallized by grafting amine group of ethylenediamine (ED) on coordinatively unsaturated Cr(III) centers, yielding a series of ED-MIL-101(Cr)-based adsorbents and their application for adsorption of U(VI) from aqueous solution were also studied. The obtained ED-functionallized samples with different ED contents were characterized by powder X-ray diffraction (PXRD), scanning electron microscope (SEM), energy dispersive X-ray spectroscopy (EDX), FTIR, elemental analysis (EA) and N_2_ adsorption and desorption isothermal. Compared with the pristine MIL-101(Cr) sorbents, the ED-functionallized MIL-101(Cr) exhibits significantly higher adsorption capacity for U(VI) ions from water with maximum adsorption capacities as high as 200 mg/g (corresponding to 100% extraction rate) at pH of 4.5 with ED/Cr ratio of 0.68 and the sorbed U(VI) ions can easily be desorbed at lower pH (pH ≤ 2.0). The adsorption mode of U(VI) ions and effects of grafted ED on the MIL-101(Cr) frameworks were also been studied by X-ray absorption spectroscopy (XAS). We believe that this work establishes a simple and energy efficient route to a novel type of functional materials for U(VI) ions extraction from solution via the post-synthetic modification (PSM) strategy.

With the rapidly increasing energy demand and uncertainty in fossil fuels-based energy sources, the use of nuclear power is predicted to continuously increase. Among these radionuclides, uranium is the predominant fuel in the nuclear reactors. As the supply of uranium from terrestrial ores is limited, extraction from other sources such as waster coal ash and seawater is actively being explored. The ocean contains about 4.5 billion tons uranium, a thousand times as much as the amount of uranium in terrestrial ores. Therefore, the ocean is an important source of uranium if it can be extracted economically^1^,^2^,^3^,^4^. On the other hand, uranium is also heaviest naturally occurring radionuclide and have various harmful effects in the environment^5^. With the rapidly development of nuclear power, large amount of waste water containing uranium are produced by the nuclear industries, ore mining. Thus it is desirable to develop novel adsorbents for extraction of uranium(VI) from seawater as well as removal from industrial waste waters and radioactive wastes^3^,^6^,^7^.

In recent years, various techniques have been studied and developed for the extraction of U(VI) from solution, including ion exchange^8^, solvent extraction^9^, co-precipitation^10^, and flotation^11^. Among these methods, adsorption is the most attractive and effective way to remove uranium from aqueous solution, due to its low cost, simple operation, and highly efficient advantages^12^,^13^. Traditional adsorbents, such as silica gels^14^, porous carbon^15^, metal oxide^16^ and polymer fibers^17^ have been widely used to extract uranium from solutions. However, due to the irregular pore size and low surface areas, these adsorbents have a low adsorption capacity for uranyl adsorption.

On account of their accessible porosity, large surface area, and adjustable pore size, metal-organic frameworks (MOFs) have shown great potential applications in gas adsorption and storage^18^, heterogeneous catalysis^19^, drug delivery^20^, and and chemical sensing^21^. Compared to conventional adsorbents, MOFs have possessed several advantages, such as mild synthetic conditions; extremely high surface areas (up to 10000 m^2^/g)^22^, which allows grafting or incorporation of exceptionally high densities of chelating ligands to bind uranyl ions; well-ordered tunable porous structures with a wide range of pore sizes, allowing the rapid diffusion of uranyl ions through nano-channels; and especially availabilities of post-synthetic modifications (PSM)^23^, by which the physicochemical properties of MOFs can be modulated. More recently, Lin *et al.*^24^ firstly reported application of MOFs (UiO-68) with phosphorylurea groups as sorbents to extract actinide elements. Shi *et al.*^25^ used LnMOFs as adsorbents for separation and enrichment of uranium in aqueous solution. These results show that MOFs with large surface areas will be good candidates for effective extraction uranium ions from aqueous solution. However, to best of our knowledge, rarely studies on the adsorption of uranium on MOFs have been reported so far, and it is still necessary to introduce organic functional groups into MOFs in order to enhance the adsorption selectivities and capacities of uranium ions.

In the present study, we report the synthesis of amine-functionallized MIL-101(Cr) by a facile coordination-based PSM strategy (Fig. 1) and their application for uranium extraction from aqueous solution. MIL-101(Cr)^26^, as a very prominent adsorbent among MOFs, is comprised of trimetric chromium(III) octahedral clusters interconnected by benzene-1,4-dicarboxylates, resulting in a highly porous three-dimensional structure. The robust MIL-101(Cr) is not only easy to synthesize but also is stable in water, common solvents, even under acidic conditions. It has two types of large mesoporous pores (29 Å and 34 Å), very large BET surface area (>3000 m^2^/g). Furthermore, after the removal of terminal water molecules on Cr(III) centers by heating in vacuum, the formed numerous active coordinatively unsaturated Cr(III) sites (CUS, up to 3.0 mmolg^−1^) can provide accessible sites for the further functionllization. As we all know, amine-based on materials exhibit good adsorption effects for metal ions extraction, especially for the U(VI) ions^27^,^28^. Ethylenediamine (ED) was chosen because if one amine group of ED is linked to a coordinatively unsaturated Cr(III) centers, the other amine group can play the role of adsorbed site to adsorb U(VI) ion from aqueous solution. In this paper, by treating the dehydrated MIL-101(Cr) with ED, we introduce the amine groups into the pores via grafting ED into the unsaturated Cr(III) centers (Fig. 1). The obtained ED-functionallized adsorbents (ED-MIL-101(Cr)) were characterized by FT-IR, PXRD, BET, SEM, and EDX. The adsorption of U(VI) from aqueous solution were performed at different pH, different organic ED contents, adsorption time, and the adsorption mode were also studied via XAS. Such a unique ED-MIL-101(Cr) functionallized materials with extremely high surface area and welled defined porosity exhibit excellent absorption performance for U(VI), which has important applications as a promising adsorbent.

## Experimental Section

### Materials and Characterization

The benzene-1,4-dicarboxylic acid (H_2_BDC) was purchased from Aldrich, chromium (III) nitrate nonahydrate (Cr(NO_3_)_3_·9H_2_O), fluorhydric acid (HF, 40%) were purchased from Sinopharm (Shanghai) Chemical Reagent Co., Ltd., China. All the other regents were commercially purchased and used as received. Standed solution of uranyl ion solution (1000 ppm) used for adsorption experiments was purchased from Analytical Laboratory, Beijing Research Institute of Uranium Geology.

The FT-IR spectra were recorded in the range 500–4000 cm^−1^ using KBr pellets on a Nicolet NEXUS 670 spectrophotometer. Elemental analysis (EA) was carried out on an Elementar Vario EI III elemental analyzer. The powder X-ray diffraction (PXRD) was recorded on X’pert PRO diffractometer at 35 kV, 25 mA for a Cu-target tube and a graphite monochromator. The morphology and chemical composition of the samples were characterized using scanning electron microscopy (SEM, JEOL JSM-6700F) equipped with an energy dispersive X-ray (Oxford Instruments INCA EDX) system. N_2_ adsorption-desorption isotherms were obtained at 77 K with liquid nitrogen on a Micromeritics ASAP 2020 surface area analyzer. Initially, the samples were vacuum degassed for 6 h at 150 °C for MIL-101(Cr) and 120 °C for samples **A**–**D** under the flow of N_2_ before the measurements. The Brunauer-Emmett-Teller (BET) specific surface areas were calculated using adsorption data in relative pressure range of P/P0 = 0.06–0.30. The pore volume was calculated by a single point method at P/P_0_ = 0.99.

The X-ray absorption spectra were collected at the beamline 14W1 of the Shanghai Synchrotron Radiation Facility (SSRF) using a Si(111) double-crystal monochromator. The electron beam energy of the storage ring was 3.5 GeV and the maximum stored current was about 210 mA. Both Cr K-edge and U L_3_-edge XAFS data were recorded in transmission mode and analyzed using standard procedures with the program Demeter^29^. Theoretical phase and amplitude functions were calculated from the program FEFF 9.0^30^.

### Preparation of ED-functionallized MIL-101(Cr) samples

MIL-101 (Cr) crystals were synthesized following the procedure reported by G. Férey^26^ with a slight modification. Typically, benzene-1,4-dicarboxylic acid (H_2_BDC) (164 mg, 1 mmol), Cr(NO_3_)_3_·9H_2_O (400 mg, 1 mmol), hydrofluorhydric acid (HF) (5 M, 0.2 mL, 1 mmol) in H_2_O (4.8 mL, 265 mmol) were mixed and heated in a Teflon-lined stainless steel autoclave at 220 °C for 8 h. After cooling to room temperature, the resulting green products were filtrated off and dried at 150 °C overnight. Then the powder was soaked in N,N’-dimethylformamide (DMF) and ultrasonicated for 2 h to remove the unreacted H_2_BDC. Finally, the filter was dried overnight in an oven at 75 °C.

To prepared the ED-functionllized ED-MIL-101(Cr) samples, the as-synthesized MIL-101(Cr) samples were firstly dehydrated at 150 °C for 12 h to remove the coordinated terminal H_2_O molecules on Cr(III) centers, so that the generated coordinatively unsaturated metal sites can serve as Lewis acid to anchor amine groups of ED. Subsequently, the hydrated powder (0.5 g) was suspended in 10 mL of anhydrous toluene. To this suspension, an appropriate amount of ED solution (50 μL for sample **A**, 100 μL for **B**, 150 μL for **C** and 200 μL for **D**, respectively) was added and the mixture was stirred with heating to reflux for 12 h. The product was recovered by filtration and washed with ethanol (15 mL × 5), and then dried overnight at room temperature in vacuum. The relative contents of ED in the functionallized samples **A**, **B**, **C** and **D** were determined by elemental analysis (see Table S1) and EDX. The molar ratios of ED to Cr in the framework for samples **A**, **B**, **C** and **D** were calculated to be about 0.34, 0.68, 0.98 and 1.42, respectively.

### Adsorption experiments

The uranyl ion adsorption experiments are performed by the batch technique in glass bottles. The pH value of the solutions is adjusted from 2.0 to 9.0 prior to the adsorption experiments by the addition of a small amount of Na_2_CO_3_/HNO_3_. About 0.05 g of dried and weighed different grafted ED-MIL-101(Cr) particles were placed in bottles that contain 0.1 L of uranyl ion solution with initial concentration of 100 ppm. The adsorption solutions are shaken at the same rate of 100 rpm at 25 °C. After being shaken for 10 h or 48 h to achieve adsorption equilibration, the particles were separated and the uranyl ion concentration in supernatant was determined by a trace uranium analyzer (WJG-III). The adsorption percentage, distribution coefficient (*K*_*d*_) and amounts of U(VI) adsorbed on the solid phase (*Q*_*t*_) were calculated as follows:


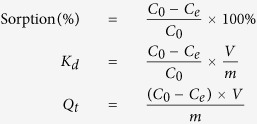


where *C*_*0*_ and *C*_*e*_ correspond to the initial and equilibrium concentrations, *V* is the volume of the suspension, and *m* is the mass of the dry adsorbent.

## Results and Discussion

### Characterization

**Figure 2:**
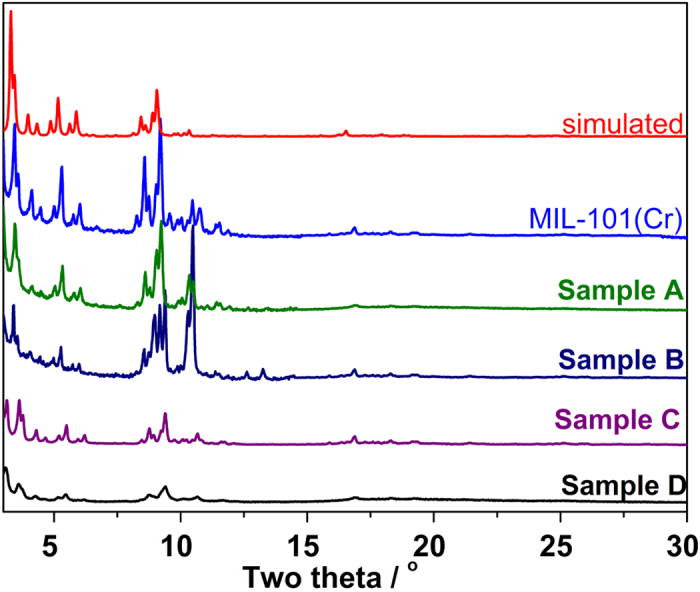
PXRD patterns of simulated, as-synthesized sample MIL-101(Cr) and different ED grafting MIL-101(Cr) A–D samples.

The synthesized structures of MIL-101(Cr) were verified by powder X-ray diffraction. As shown in Fig. 2, the PXRD patterns of our synthesized MIL-101(Cr) matched well with those reported in the literature confirming the formation of MIL-101(Cr)^26^. After subsequent ED grafting steps, the ED-functionallized samples show almost similar patterns with the pristine MIL-101(Cr), confirming the structures of MIL-101 (Cr) retained intact with no apparent loss of crystallinity, but with some slight decreases of peak intensities with the increase of ED contents, due to the partial filling by the grafting ED molecules.

**Figure 3:**
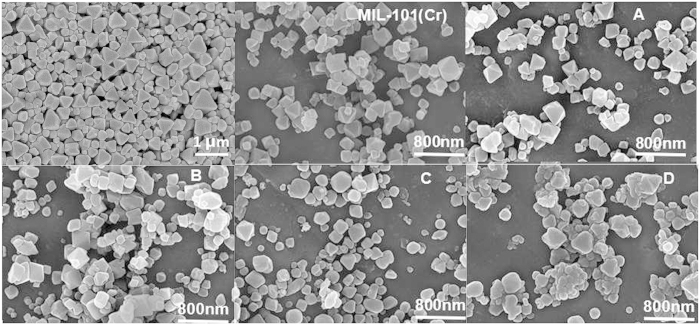
SEM images of the as-synthesized MIL-101(Cr) crystals and ED-MIL-101(Cr) (A–D) samples.

The SEM images of the pristine MIL-101(Cr) crystals are discrete octahedron with a smooth surface and have an average of 300 nm. However, after the ED functionallization, the octahedral morphologies gradually disappeared and the surface of the ED-functionallized MIL-101(Cr) samples **A**, **B**, **C** and **D** tender to be rougher after functionallization, which is consistent with the PXRD data. Moreover, the ED-functionallized **D** sample, increasing ED/Cr molar ratio to 1.42, caused a significant morphological change from regular octahedron to irregular particles (Fig. 3). The EDX spectra of the ED-functionallized samples revealed that the samples are composed of C, O, F, Cr and N (the Al and Pt elements are from the SEM preparation procedure). The relative contents of N in the functionallized samples **A**, **B**, **C** and **D** were determined to be about 2.81, 5.72, 8.17 and 11.84 mmol g^−1^, which correspond to ED/Cr ratio of 0.34, 0.68, 0.98 and 1.42, respectively (See Figure S1), which is consistent with the elemental analysis, suggesting that content of amine grafted on the framework can easily be tuned by varying molar ratio of ED to the framework. Moreover, with the increases of ED contents, the color of the samples also changes from green (MIL-101(Cr)) to brown (See Figure S2).

The IR spectra of the pristine MIL-101(Cr) and different ED grafting MIL-101(Cr) confirm the grafting ED on MIL-101 (Cr) (See Figure S3). Compared with the pristine MIL-101(Cr), the observed representative peaks between 2800–3000 cm^−1^ and the peaks at about 1050 cm^−1^, which can be attributed to the ν_CH_ and ν_CN_ stretching vibrations, respectively, confirming that the expected grafted ED materials via post-synthetic modifications on the unsaturated Cr(III) are successfully obtained. It is worth noting that these characteristic peaks become stronger with the increase of ED ratio in MIL-101(Cr).

**Figure 4:**
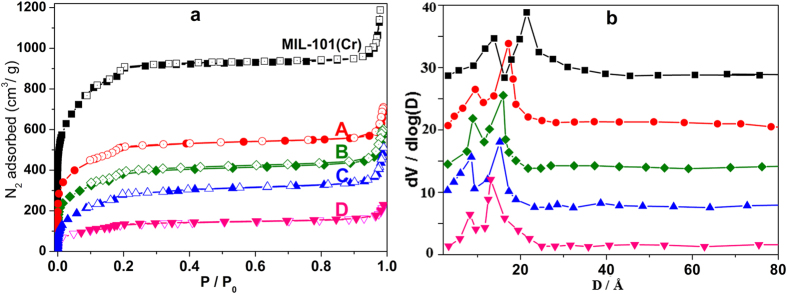
(**a**) N_2_ adsorption-desorption isotherms at 77 K for pristine MIL-101(Cr), and different ED contents grafting ED-MIL-101(Cr) of **A**, **B**, **C** and **D** samples; (**b**) the corresponding pore-size distribution curves.

The resulting pore modifications are also visible in the N_2_ adsorption isotherms of the ED-grafted MIL-101(Cr). The N_2_ adsorption isotherms, BET surface areas and pore volumes are shown in Fig. 4. N_2_ adsorption measurements of the pristine MIL-101(Cr) showed a high surface area of the obtained porous material *S*_BET_ = 2852 m^2^ g^−1^. Compared with the pristine MIL-101(Cr), the grafted ED-MIL-101(Cr) materials exhibit a significant decrease of the N_2_ adsorption amounts and the corresponding BET surface areas also decrease from 2852 m^2^ g^−1^ to 517 m^2^ g^−1^ with the increase of grafted ED contents, which may be due to substitution of H_2_O by ED with larger size. The pore volumes and pore size are also decreased gradually with the increase of grafted ED contents (Table 1). Due to the terminal water molecules on Cr(III) centers protrude towards the center of the cages, the grafted ED substituted of the terminal H_2_O molecules also presented mainly at the center of mesoporous cages, which maybe convenient for the coordination of ED with U(VI) ions and therefore lead to the high adsorption capacities.

### U(VI) sorption studies

**Figure 5:**
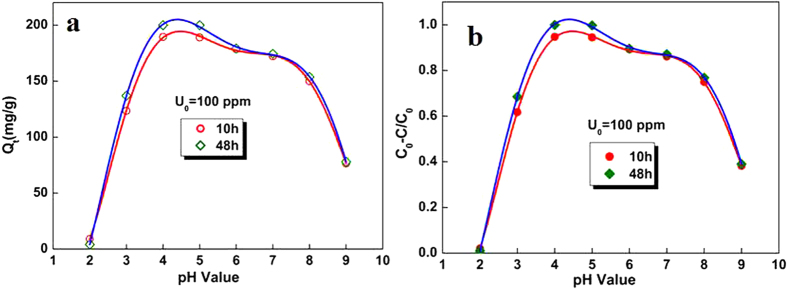
Effect of pH value on adsorption of U(VI) ions. Initial uranium concentration U_0_ = 100 ppm; pH = 2.0–9.0; T = 25 °C; t = 10 h and 48 h.

The pH value is one key parameter for the adsorption of metal ions because of the hydratation and complex formation of metal ions and the study on the effect of the pH value can also help us to determine the desorption conditions. The influence of pH on the adsorption of U(VI) on ED-MIL-101(Cr) particles were investigated at pH = 2.0–9.0. As shown Fig. 5, the U(VI) adsorption of ED-MIL-101(Cr) particles is strongly dependent on the pH of the solution, which influences the existence form of U(VI) and properties of functional groups of adsorbents: at low pH (pH = 2.0) solution, the U(VI) adsorption can be negative. The U(VI) adsorption capacity increases dramatically with increasing pH and to a maximum value (200 mg/g) at about pH = 4.5, corresponding to nearly 100% U(VI) ions being extracted from solution. Then a slight decrease is observed with further increase of pH value and decline more rapidly when pH value higher than 8.0. This phenomenon illustrated that the desorption procedure can be carried out in low acidic solution (pH ≤ 2.0). The existence form of U(VI) in aqueous solutions is extremely complicated: U(VI) mostly exists as UO_2_^2+^ in its hydrolysis complexes, carbonate complexes and multinuclear hydroxide as a function of pH and concentration under experimental conditions^31^. UO_2_^2+^ was the manly species in acidic pH from 2.0 to 5.0, especially when the pH value is 2.0^32^,^33^. When the pH was from 5.0 to 8.5, the UO_2_^2+^, [UO_2_(OH)]^+^, [(UO_2_)_3_O(OH)_3_]^+^, [(UO_2_)_2_(OH)_2_]^2+^ and [UO_3_(OH)]^5+^ coexist^33^,^34^. At a pH value higher than 9.0, tricarbonate uranium complex [UO_2_ (CO_3_)_3_]^4−^ plays as the dominant form. The amine is found to be less effective for complexion UO_2_^2+^ in the competition with CO_3_^2−^, which result in the lower adsorption ratio. On the other hand, the protonation of amine groups greatly depend on pH value of solution, where the lone pair electrons on N were occupied by hydrogen, which made coordination of amine inert in strong acid condition (pH ≤ 2.0).

**Figure 6:**
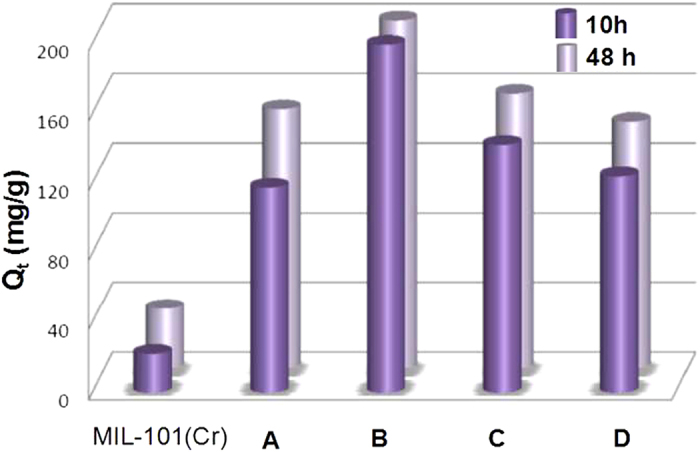
Effect of ED dosage on adsorption of U(VI) ions. Initial uranium concentration U_0_ = 100 ppm; pH = 4.5; T = 25 °C; t = 10 h and 48 h.

The effect of the grafted ED dose of MIL-101(Cr) on the adsorption of U(VI) was also studied (Fig. 6). Five adsorbents containing different grafting ED contents were added to uranium (VI) solution by keeping other parameters constant. Compared with pristine MIL-101(Cr), the introduce of ED on MIL-101(Cr) evidently enhances the uptake of U(VI), and with increasing ED amount, the adsorption capacities increase rapidly and reach to maximum with ED/Cr ratio being 0.68 (corresponding to two open sites of each trimetric Cr(III) cluster are both occupied by ED molecules) with adsorption amount of 200 mg/g, corresponding to 100% adsorption rate. The adsorption capacities decrease with the further increase of ED grafted on MIL-101(Cr) frameworks (Table 1). The increase in adsorption efficiency with the amount of ED can be attributed to increase of adsorption sites. With the further increase of ED leading to aggregation, which partly blocked the pores of pristine MIL-101(Cr) materials, the total surface area of ED-MIL-101(Cr) decreases further, which decrease the diffusion of uranyl ions through nano-channels. The recycling studies were also performed under optimal conditions. After four cycles, there is a ca. 58% reduction of sorption capacity compared to the fresh synthesized sample **B**, which maybe due to the decrease ratio of ED on the porous MIL-101(Cr) materials. This is contributed to the partial substitution of ED on Cr(III) centers by H_2_O molecules under desorption process (low pH solution), but frameworks of samples are stable, which can be easily functionalized for further sorption (Figure S6).

### X-ray Absorption Spectroscopy (XAS)

Synchrotron radiation X-ray absorption near-edge structure (XANES) data were performed to investigate the local coordination environments around the Cr sites of ED functionalized MIL-101(Cr) materials before and after the adsorption of U(VI). The XANES features mainly originate from photoelectron multi-scattering contributions, being strongly sensitive to the local structures around absorbers. The effect of ED content on the local structure around Cr atoms is evident from the comparison of the spectra shown in Fig. 8a. Both spectra have been normalized in the energy range 5970–6140 eV. Two significant structural regions need to be mentioned. (1) Both spectra have subtle pre-peak feature, which corresponded to a transition to 4p states hybridized with the 3d band induced by distorted octahedral structure. With the increase of ED ratio, no obvious changes can be seen in the pre-peak regions, which imply that the valence state of Cr(III) maintains in all samples. (2) Significant change occur in the main peak of Cr K-edge XANES spectrum upon increasing the ED ratio, that is, the intensity of main peak decrease and replaced by an extremely broaden peak. According to the dipole selection rules, electronic transitions of the main peak at the Cr K-edge XANES pattern are dominated by transitions from 1 s to 4p empty states. When coordinately unsaturated Cr(III) sites are gradually occupied by the amine of ED molecules, such coordinated symmetry around Cr atoms decrease, the degenerate of p orbit state decrease, and thus evidently broaden the main peak in the Cr K-edge XANES spectrum. Such results are exactly consistent with the analysis of N_2_ adsorption isotherms.

**Figure 7:**
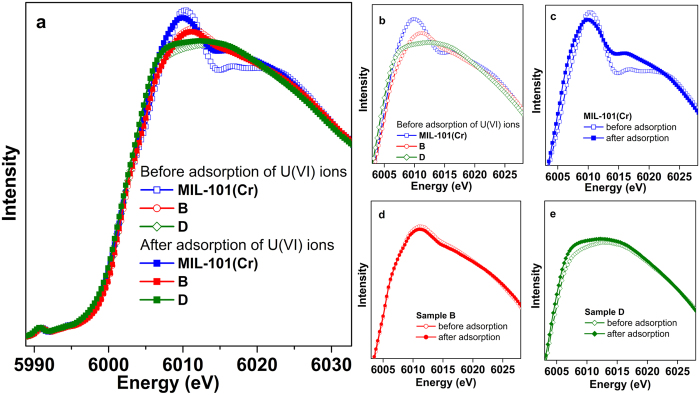
(**a**) Comparison of experimental Cr K-edge XANES spectra for different samples before and after the adsorption of U(VI); (**b**–**e**) Magnification of experimental white-line region of Cr K-edge XANES for different samples.

As shown in Fig. 7b–e, we selectively magnify and compare the Cr K-edge XANES data in different sample before and after the adsorption. Compared to the ED grafting MIL-101(Cr) samples, the biggest differences of pattern appear in the pristine MIL-101(Cr), which imply possible different adsorption mode in these samples. According to previous analysis, in the pristine MIL-101(Cr) main adsorption of U(VI) origins in the mesopores of the frameworks, namely physical adsorption, which occur near the Cr(III) center and thus strongly affect the Cr K-edge XANES pattern as shown in Fig. 7c. For sample **B** shown in Fig. 7d, the influence of U (VI) adsorbed for the Cr(III) coordination environment is negligible, due to the large amount of U(VI) are coordinated to amine of grafted ED as expected. The influence of U-adsorption on the Cr(III) center in the sample **D** shown in Fig. 7e is in between, in which the U(VI) ions are not only adsorbed chemically by amine of the grafted ED on Cr(III) centers, but slightly physically by the mesopores as expected. Quantitative information about the local structures was further obtained by fitting EXAFS data shown in Figure S4 and data are extracted in Table S2. In all samples, no obvious changes of bond length can be seen. But visible changes occur in the coordination number in the pristine MIL-101(Cr) and the sample **D**, while same metric parameters can be obtained in the sample **B** before and after the U(VI) adsorption. Such results are consistent with the XANES analysis.

**Figure 8:**
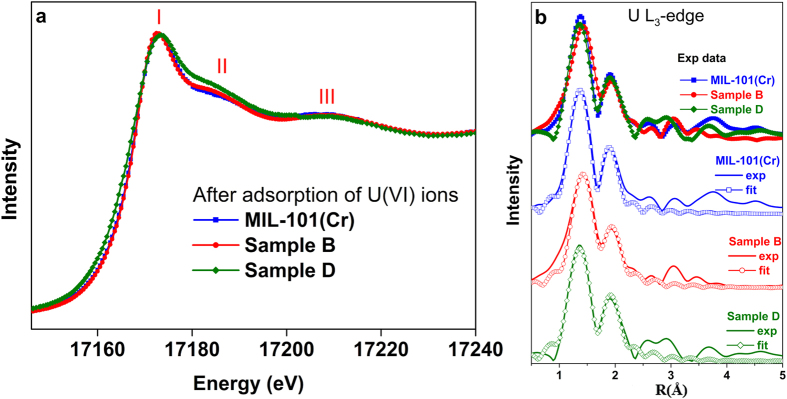
(**a**) Comparison of experimental U L_3_-edge XANES spectra for pristine MIL-101(Cr), and different ED contents grafting ED-MIL-101(Cr) samples after the adsorption of U(VI). (**b**) Experimental Fourier Transform at of the U L_3_-edge EXAFS data for different samples and their corresponding fits.

We also study the local structure around the U(VI) ions in different ED-functionallized-MIL-101(Cr) samples after adsorption. In the Fig. 8a, similar XANES pattern can be seen in all U-absorbed MIL-101(Cr) samples with or without ED-grafting, including a strong main peak (I), a shoulder II at about 15 eV and feature III at about 35 eV above the main peak maximum, which implies that the bipyramid skeletal structure of uranyl was maintained. Main feature changes appear in the regions of peak II/III with the increase of ED ratio. According to previous work^35^,^36^, the energy positions of two continuum resonance peaks II and III depend on the U-O_ax_ and U-O_eq_ distances, respectively. Quantitive bondlength information can be extracted by EXAFS fits. In *R* space of Fig. 8b, the peaks at ~1.3 eV and ~2 eV are assigned to the single scattering paths of oxygen (axial), ligand (equatorial). Considering the large errors of coordination number (CN) (10%~25%) in the EXAFS fits and strong relationship between the CN and disorder, during the fit procedure we more care about the information of bond length and thus the number of axial and equatorial coordinated atoms was fixed to two and six, respectively. In Table 2, obvious bondlength lengthen in sample **B**, possibly induced by strong interaction in the equatorial plane. According to references^37^,^38^, the charge transfer of amine to U(VI) are stronger than water molecule to U(VI), and thus can push the axial oxygen atoms away from the U(VI) center. It is reasonable explanation because in sample **B** U(VI) are mainly coordinated to the amine groups, while in pristine MIL-101(Cr) and sample **D** physical adsorption are dominant.

## Conclusion

In conclusion, the present work demonstrates a facile strategy to fabricate ED-functionallized porous MIL-101(Cr) as a novel type of adsorbent for removal of U(VI) ions from solution. ED-MIL-101(Cr) samples with different ED/Cr molar ratio were prepared by ED grafting on coordinatively unsaturated Cr(III) center in a porous MIL-101(Cr) particles, and their structures, morphologies and porosity were characterized. Compared to the pristine MIL-101(Cr) samples, the ED-functionallized samples exhibited a high highly efficient in adsorbing U(VI) ions, with the maximum adsorption capacities as high as 200 mg/g (corresponding to 100% extraction rate) at pH of 4.5, which can also easily desorbed using a solution with pH ≤ 2.0. When the grafted ED/Cr ratio is 0.68, the sample exhibits the maximum adsorption capacity.

We believe that this work establishes a simple and energy efficient route to a novel functionallized materials for U(VI) ions extraction on the basis of the porous functionallization of MOFs-based materials. Also, the post-synthetic modification (PSM) strategy will offer a plethora of opportunities for functional MOFs-based porous materials.

## Additional Information

**How to cite this article**: Zhang, J.-Y. *et al.* Adsorption of Uranyl ions on Amine-functionalization of MIL-101(Cr) Nanoparticles by a Facile Coordination-based Post-synthetic strategy and X-ray Absorption Spectroscopy Studies. *Sci. Rep.* **5**, 13514; doi: 10.1038/srep13514 (2015).

## Supplementary Material

Supplementary Information

## Figures and Tables

**Figure 1:**
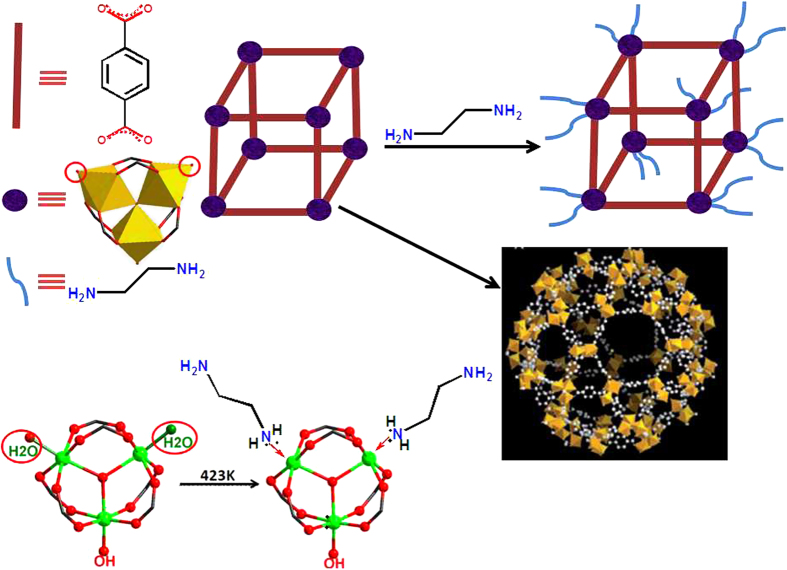
The work road-map of the ED-functionallized MIL-101(Cr) through the post-synthetic modifications.

**Table 1:** The U(VI) adsorption data of MIL-101(Cr) and its ED-functionallized samples with different ED contents.

Samples	S_BET_ (m^2^/g)	V_t_ (cm^3^/g)	ED contents (mmol/g)	ED/Cr ratio	ED/Cr_CUS_ratio	Q_t_(mg U/g)^a^
MIL-101(Cr)	2852	1.32	–	–	–	22.13/34.38
Sample **A**	1821	1.04	1.404	0.34	0.505	117.78/149.1
Sample **B**	1352	0.69	2.86	0.68	1.03	199.7/200
Sample **C**	1141	0.57	4.09	0.98	1.47	142.24/158.02
Sample **D**	517	0.34	5.92	1.42	2.13	124.32/141.92

^a^The value are obtained under U_0_ = 100 ppm, pH = 4.5, T = 25 °C, t = 10 h and 48 h, respectively.

**Table 2:** Result of the U L_3_-edge EXAFS fit of different ED grafting MIL-101(Cr) after adsorption of U(VI) ions.

Samples	Bond Type	N	*R*(Å)	σ^2^ × 10^−3^ (Å^2^)	*R* factor
MIL-101(Cr)	U-O_ax_	2.0	1.78 ± 0.02	2.0 ± 0.4	0.01
	U-O_eq_	6.0	2.40 ± 0.02	12.7 ± 1.5	
Sample **B**	U-O_ax_	2.0	1.80 ± 0.02	3.2 ± 0.8	0.01
	U-O_eq_	6.0	2.40 ± 0.02	14.5 ± 1.6	
Sample **D**	U-O_ax_	2.0	1.77 ± 0.02	2.5 ± 0.6	0.01
	U-O_eq_	6.0	2.40 ± 0.02	12.8 ± 0.9	
