# Supplementary material for: Adsorption of Uranyl ions on Amine-functionalization of MIL-101(Cr) Nanoparticles by a Facile Coordination-based Post-synthetic strategy and X-ray Absorption Spectroscopy Studies
Source: Sci Rep. 2015 Sep 10;5:13514. doi: 10.1038/srep13514 (PMC4564734; doi:10.1038/srep13514)

**Supporting Information**

**Adsorption of Uranyl ions on Amine-functionalization of MIL-101(Cr) Nanoparticles by a Facile Coordination-based Post-synthetic strategy and X-ray Absorption Spectroscopy Studies**

Jian-Yong Zhang^†,1,2^, Na Zhang^†,1^, Linjuan Zhang^3^, Yongzheng Fang^1^,*, Wei Deng^1^, Ming Yu^2^, Ziqiang Wang^2^, Lina Li^4^, Xiyan Liu^2^ and Jingye Li^2^,*

Table S1. Elemental analysis of MIL-101(Cr), different ED contents grafting MIL-101(Cr) of **A**, **B**, **C** and **D** samples.

|  | N | C | H |
| --- | --- | --- | --- |
| Theoretical MIL-101(Cr) | 0 | 37.27 | 2.87 |
| MIL-101(Cr) | 0.15 | 37.51 | 2.54 |
| Dehydrated MIL-101(Cr) | 0.26 | 42.79 | 2.11 |
| Sample **A** | 3.56 | 42.38 | 2.45 |
| Sample **B** | 7.03 | 42.25 | 3.42 |
| Sample **C** | 9.79 | 41.22 | 4.01 |
| Sample **D** | 12.93 | 41.46 | 4.67 |

Table S2. Result of the Cr K-edge EXAFS fit of different ED grafting MIL-101(Cr) with and without adsorption of U(VI) ions. *N* is the coordination number. *R* is the distance between the uranium adsorber and surrounding coordination atoms. σ^2^ is the mean square disorder.

| Samples | Bond Type | *N* | *R*(Å) | σ^2^×10^-3^(Å^2^) | *R* factor |
| --- | --- | --- | --- | --- | --- |
| MIL-101(Cr)-before | Cr-O | 6.0±0.2 | 1.97±0.02 | 3.5±0.4 | 0.01 |
| MIL-101(Cr)-after | Cr-O | 6.1±0.3 | 1.97±0.02 | 3.5±0.7 | 0.01 |
| Sample **B**-before | Cr-O | 5.9±0.3 | 1.97±0.02 | 3.9±0.5 | 0.01 |
| Sample **B**-after | Cr-O | 5.9±0.2 | 1.97±0.02 | 3.9±0.4 | 0.01 |
| Sample **D**-before | Cr-O | 5.7±0.2 | 1.97±0.02 | 4.4±0.4 | 0.01 |
| Sample **D**-after | Cr-O | 5.9±0.3 | 1.97±0.02 | 4.4±0.6 | 0.01 |

Figure S1. The EDX spectra of the grafted different ED-MIL-101(Cr) **A**-**D** samples.


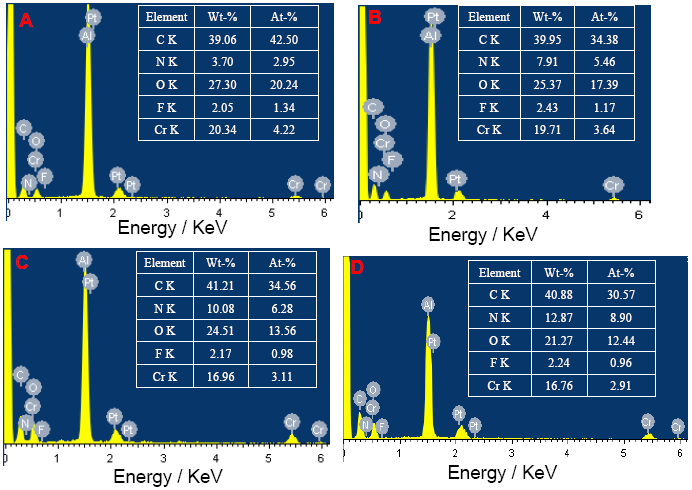


Figure S2. The photos of pristine MIL-101(Cr), dehydrated MIL-101(Cr) and the grafted different ED-MIL-101(Cr) **A**-**D s**amples.


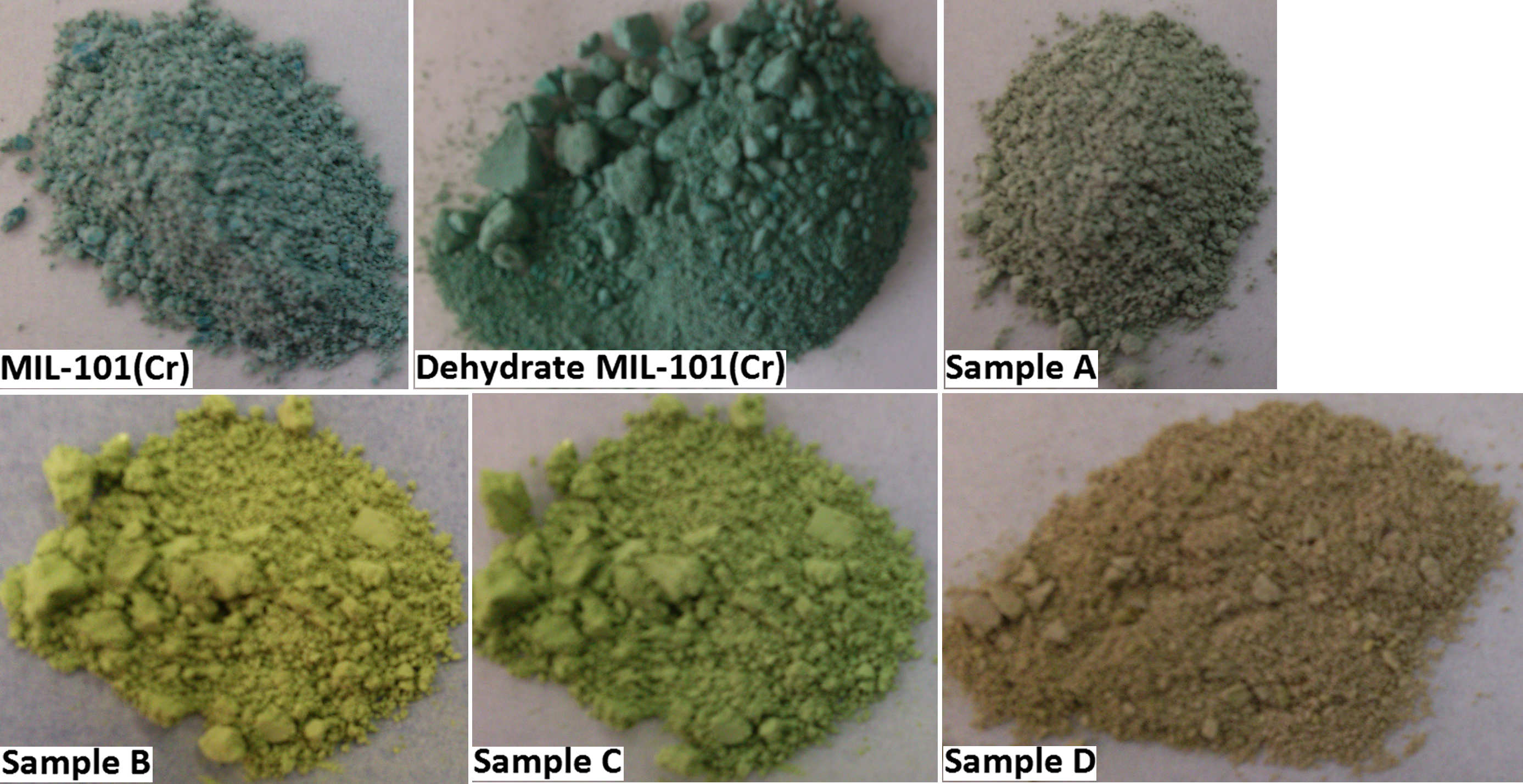


Figure S3. The FT-IR spectra of the MIL-101(Cr) and different ED contents grafting ED-MIL-101(Cr) samples.


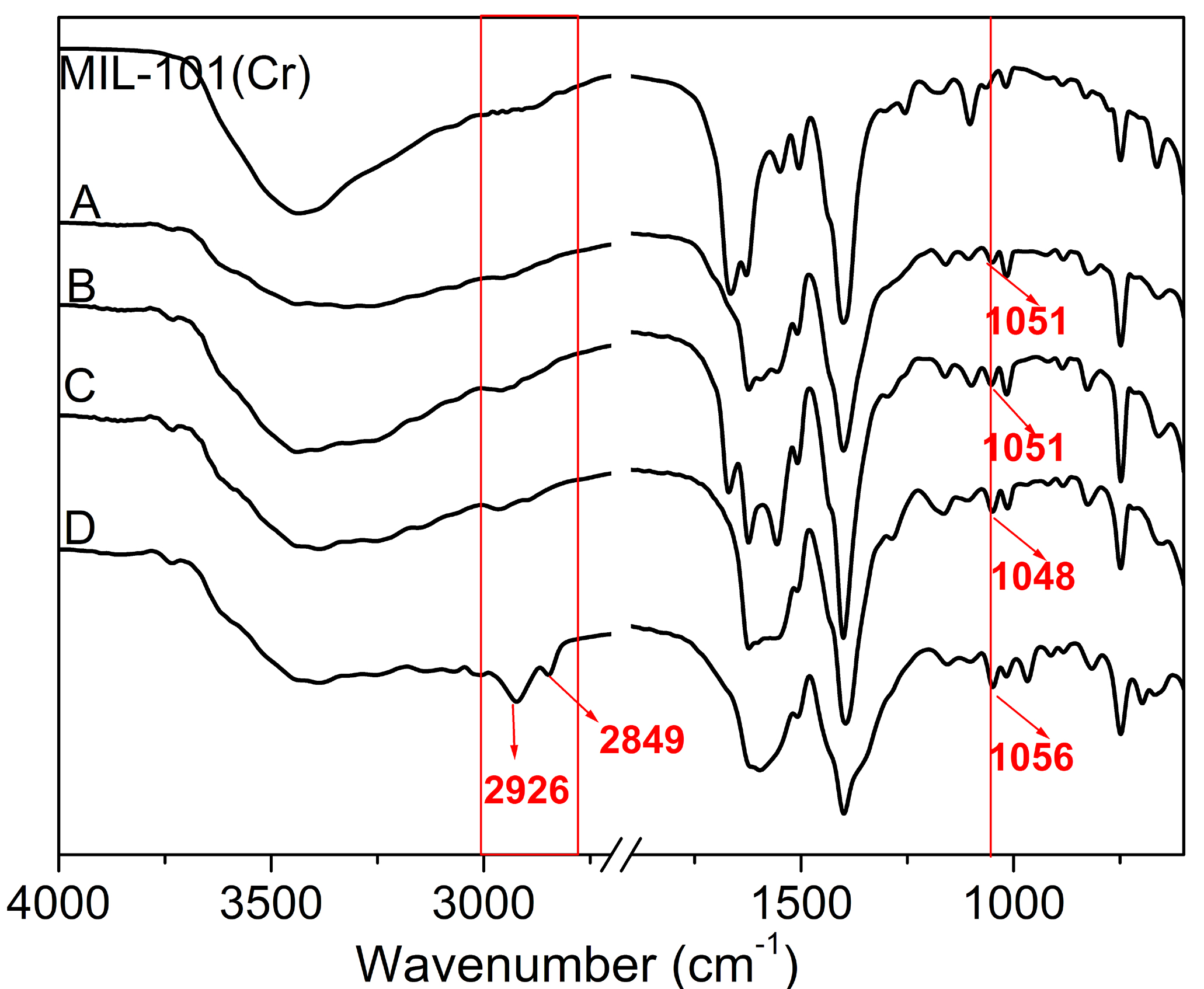


Figure S4. The TGA plots pristine MIL-101(Cr), and different ED contents grafting ED-MIL-101(Cr) of **A**, **B**, **C** and **D** samples.


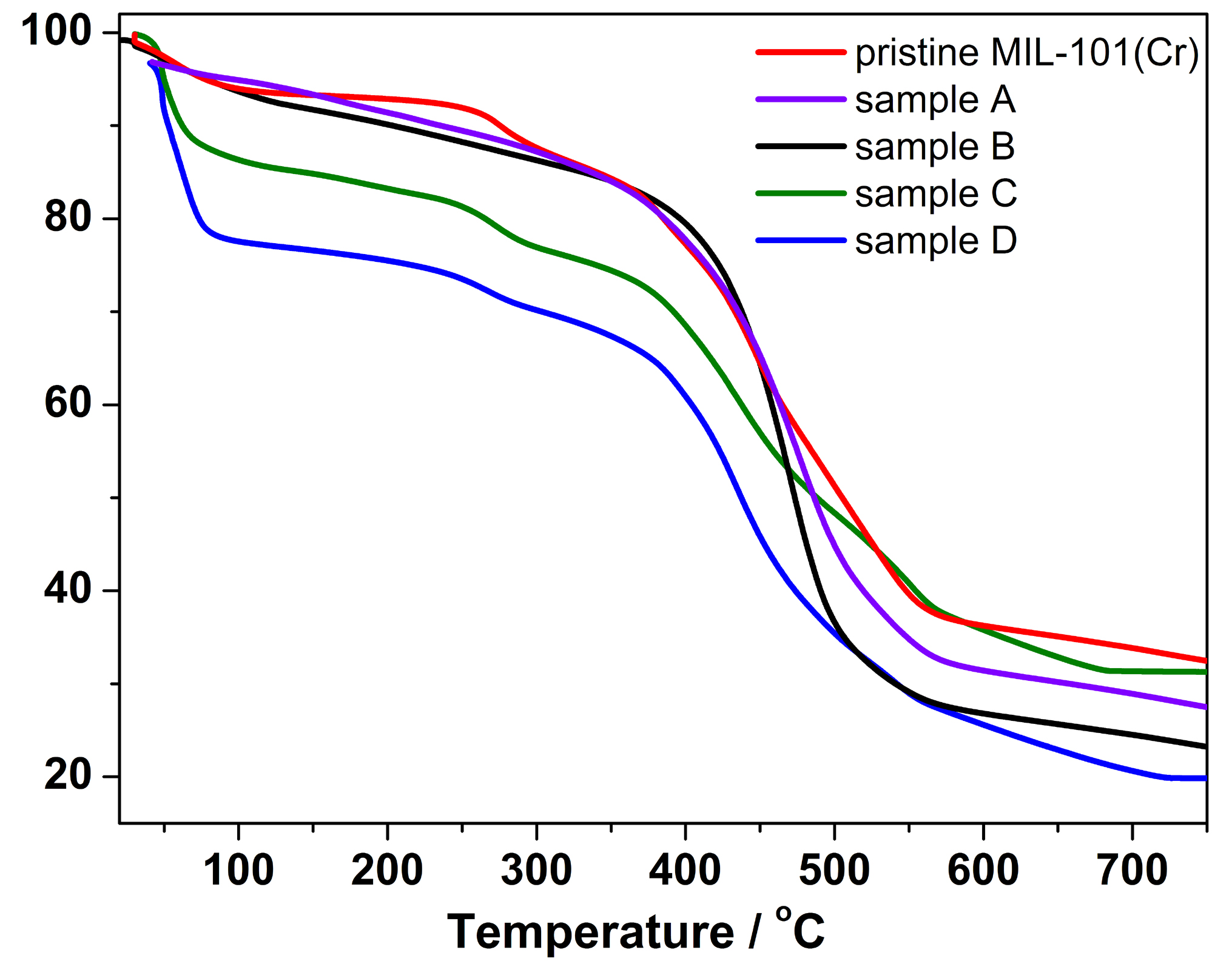


Figure S5. (a) Recycling experiments of sample **B** adsorbent. Initial uranium concentration U_0_=100 ppm; pH=4.5; T=25^o^C; t= 48h; (b) The PXRD patterns of the sample **B** after four runs.


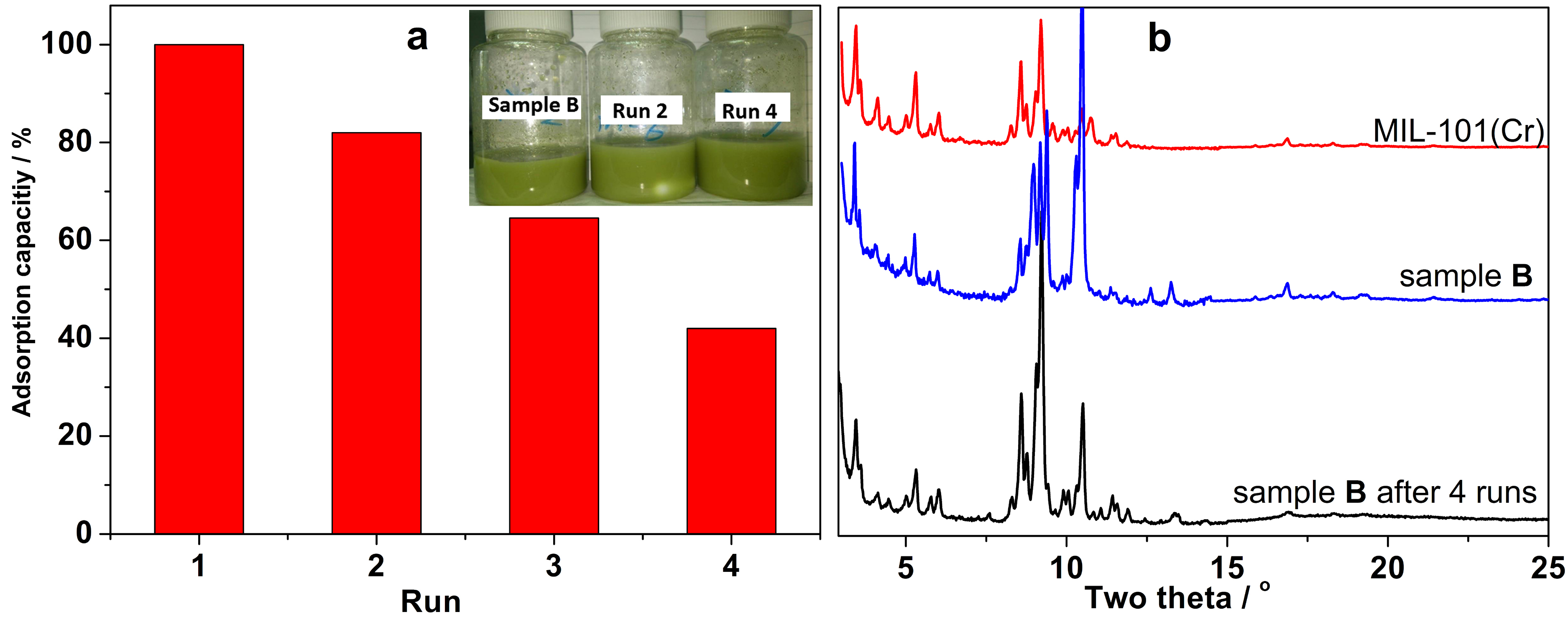


Figure S6. Experimental Fourier Transform of the Cr K-edge extended X-ray absorption fine structure (EXAFS) data for pristine MIL-101(Cr), **B** and **D** samples and their corresponding fits.


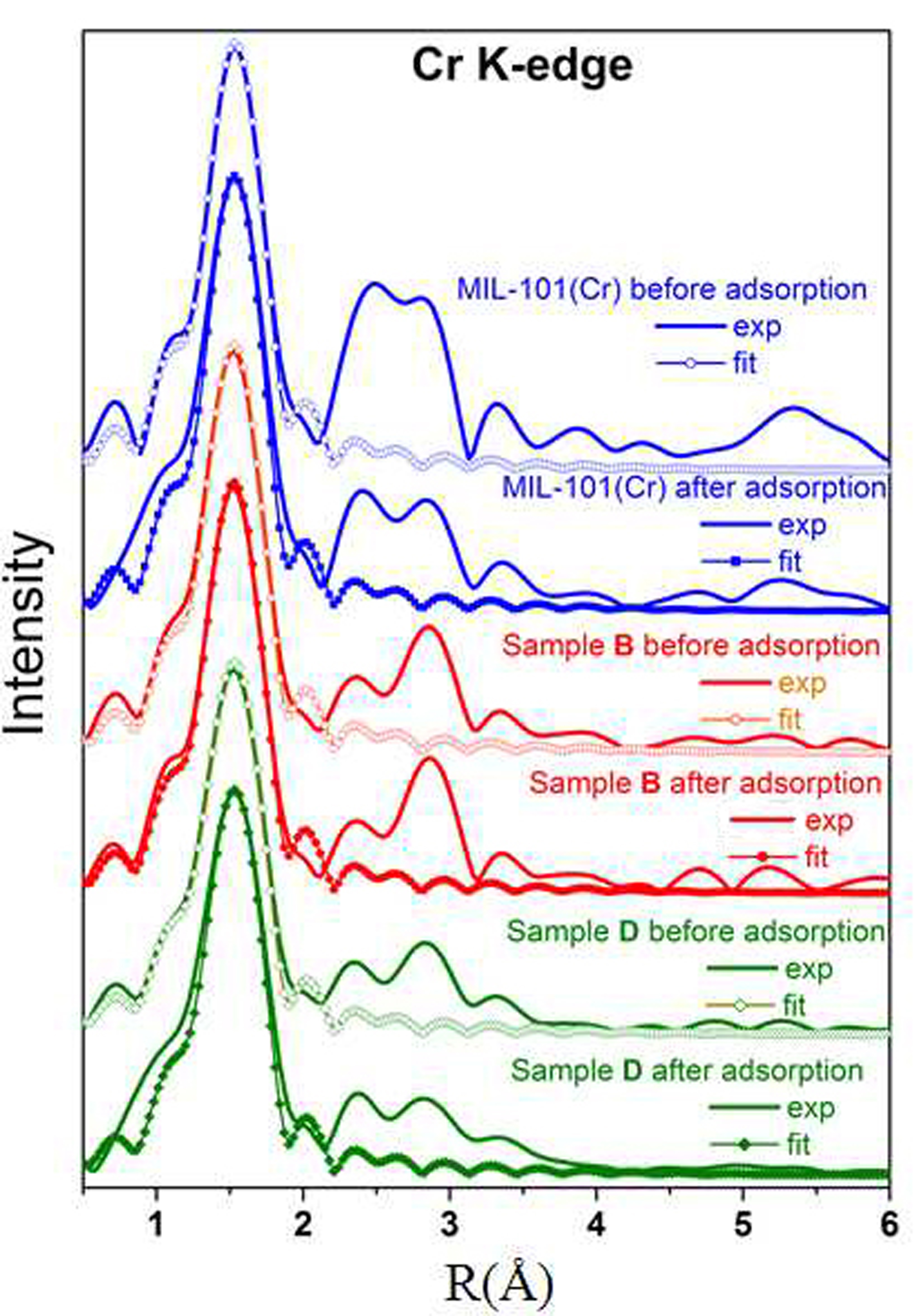

Supplement: Supplementary Information [file srep13514-s1.doc]
